# Towards understanding microvillus inclusion disease

**DOI:** 10.1186/s40348-016-0031-0

**Published:** 2016-01-29

**Authors:** Georg F. Vogel, Michael W. Hess, Kristian Pfaller, Lukas A. Huber, Andreas R. Janecke, Thomas Müller

**Affiliations:** Department of Paediatrics I, Medical University of Innsbruck, Anichstrasse 35, 6020 Innsbruck, Austria; Division of Cell Biology, Biocenter, Medical University of Innsbruck, Innsbruck, Austria; Division of Histology and Embryology, Medical University of Innsbruck, Innsbruck, Austria

**Keywords:** MVID, Microvillus inclusion disease, Enteropathy, MYO5B, STX3

## Abstract

Microvillus inclusion disease (MVID) is characterised by onset of intractable life-threatening watery diarrhoea during infancy. Transmission electron microscopy demonstrates shortening or absence of apical microvilli, pathognomonic microvillus inclusions in mature enterocytes and subapical accumulation of periodic acid-Schiff-positive granules or vesicles confirming diagnosis. Mutations in *MYO5B* have been found to cause MVID. In two patients with MVID, whole-exome sequencing of DNA revealed homozygous truncating mutations in *STX3*. Mutations in these genes disrupt trafficking between apical cargo vesicles and the apical plasma membrane. Thus, disturbed delivery of certain brush border membrane proteins is a common defect in MVID.

## The history of microvillus inclusion disease

In 1978, Davidson and colleagues first described five infants with severe diarrhoea from birth and failure to thrive [1]. The disease was identified as a congenital enteropathy marked by villus atrophy, severe diarrhoea with partial sodium loss and malabsorption. Furthermore, jejunal biopsies displayed in electron microscopy (EM) cytoplasmic inclusions with brush border microvilli on their inside. Over the following years, the disease was given several names: Davidson disease [1], congenital familial protracted diarrhoea with enterocyte brush-border abnormalities, congenital microvillus atrophy [2] and microvillus inclusion disease (MVID). The latter one was shaped by Cutz and colleagues in 1989 [3]. This study sets the diagnostic standard, already discussing further possible mechanisms for the formation of microvillus inclusions (ectopic brush border formation at intracellular sites [3] versus engulfment of brush border via autophagy/macropinocytosis [1]; see also below the paragraph on pathophysiology). The following years, an increasing number of patients were diagnosed with MVID and diagnostic criteria were refined [4–6]. In 2008, mutations in *MYO5B* were identified as causal for MVID [7]. This finding initiated further research trying to unravel the pathophysiology of MVID and the specific function of the motor protein Myo5B in polarised epithelial cells [8–10]. With the identification of mutations in a second gene, *STX3*, causative for MVID, molecular and genetic analyses gained pace pushing MVID to the ‘centre stage’ of molecular paediatric research.

## Clinical features of MVID

MVID patients typically present intractable watery diarrhoea, leading to a severe loss of body weight, and metabolic acidosis due to bicarbonate loss [1, 3, 4, 11]. Early-onset MVID that starts within the first days after birth can be discriminated from late-onset MVID cases [4, 12]. The latter one becomes clinically apparent only at the age of 2 to 3 months. In general, pregnancy was reported to be without complications, but occasionally, maternal polyhydramnios is present. Diarrhoea is the main, often life-threatening symptom of MVID. Stool volumes range between 150 and 300 ml/kg/day and respond only slightly to bowel arrest [4].

In most cases, no additional clinical signs, such as malformations, or other organ manifestations accompany MVID. However, cholestatic liver disease might be present in up to one third of patients [13], and cases of associated renal Fanconi syndrome [14] have been reported.

Treatment aims at supplementing the fluid and nutrient loss. Thus, life-long total parenteral nutrition (TPN) is generally required. No causative cure is available, but small bowel transplantation (intestinal Tx) is able to cure the severe diarrhoea. Both TPN and intestinal Tx come with side effects, such as cholestasis or infections caused by the immunosuppressive regime.

The limited therapeutic options and the severity of the disease often lead to the patients’ death within the first 3 years. However, patients with late-onset MVID sometime tolerate enteral feeding. The requirement of parenteral nutrition can be reduced to once or twice per week [4, 6].

## Genetics

Since the identification of mutations in the *MYO5B* gene in 2008, an increasing number of mutations causing MVID are described in the literature [7, 8, 12], thereby confirming autosomal recessive inheritance of MVID. Homozygous (mostly originating from consanguine parents) and compound-heterozygous missense and nonsense mutations (mostly from non-consanguine parents) were reported. However, in some patients, only one heterozygous mutation and no second mutation could be identified [12] upon sequencing all exons and exon-intron boundaries. With this technique, deep intronic mutations, mutations in regulatory regions, and deletions and duplications involving whole exons cannot be revealed and may be missed. Nowadays, commercial multiplex ligation-dependent probe amplification (MLPA) analysis is available to detect larger intragenic deletions and duplications. Sequencing and MLPA analysis detect biallelic mutations in >90 % of patients referred with a clinical/histopathologic diagnosis of MVID (*n* = 70, our unpublished data). Van der Velde and colleagues have methodically characterised all currently reported mutations in MYO5B and linked them to potentially impaired functions of the different domains of the actin motor protein MYO5B [12].

The second gene in which MVID causing mutations were identified was *STX3* [15]. So far, two patients of 2 years of age have been reported, both with nonsense mutations resulting in truncations of the apically targeted *N*-ethylmaleimide-sensitive factor attachment protein receptor (SNARE) protein syntaxin3 [15]. Genotype-phenotype correlation with respect to MYO5B and STX3-related MVID has not been reported so far, due to the small number and young age of patients with STX3 mutations.

## Diagnosis

**Fig. 1:**
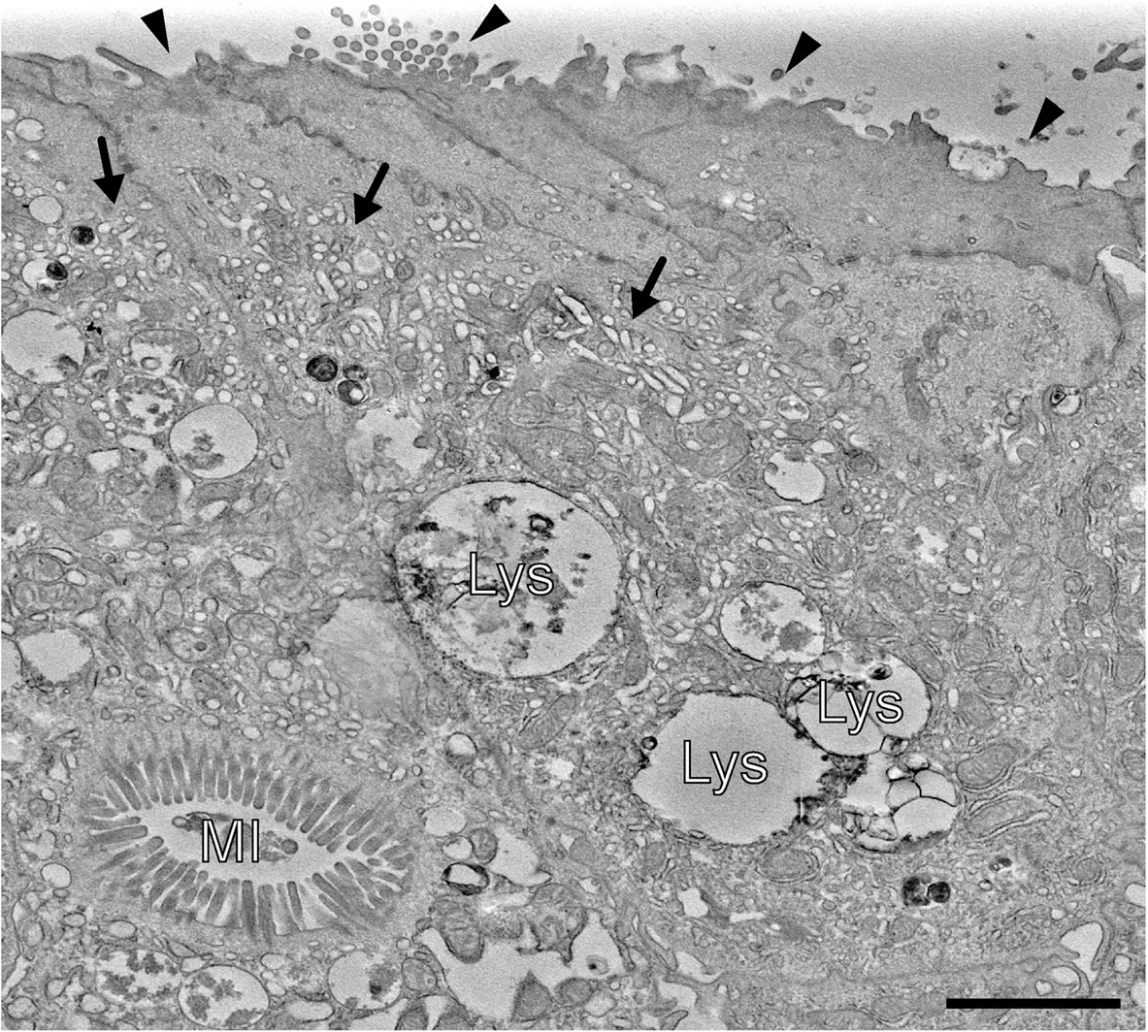
Electron micrograph of patient’s duodenal enterocytes depicting the three ultrastructural hallmarks of MVID (homozygous c.1323–2A > G splice-site mutation in *MYO5B*). *Black arrow heads* mark the shortening or loss of apical microvilli. Subapical accumulations of tubulo-/vesicular structures (secretory granules) are marked by *black arrows. MI* intracellular microvillus inclusion, *Lys* lysosomes. *Scale bar* = 2 μm

The clinical presentation of MVID is that of severe diarrhoea, most often starting within the first week of life. The diarrhoea has a secretory component, which persists even when giving nil per mouth and does not clinically resolve upon elimination of dietary components. Villus atrophy, brush border reduction and increased periodic acid-Schiff (PAS) staining throughout the subapical cytoplasm are often found in light microscopy. Immunohistochemistry for brush border components CD10 (a neutral membrane-associated peptidase) [6, 16], Villin [17] and Rab11a [18, 19] shows an irregular, broadened apical signal, corresponding to abnormal subapical localization of brush border components, which strongly raises suspicion of MVID. Analysis of small intestinal biopsies by EM remains the best tool for histological diagnosis of MVID. EM reveals shortening or absence of brush border microvilli, so-called microvillus inclusions (vacuoles bearing centripetal microvilli in about 10 % of small gut villus enterocytes), and a subapical accumulation of different kinds of vesicular/tubular structures, referred to as ‘PAS positive secretory granules’ [5]—the three ultrastructural/diagnostic hallmarks of MVID (compare Fig. 1). Differences in the abundance of these features are often observed between villus and crypt enterocytes. While villus enterocytes may display all three hallmarks, crypt enterocytes generally display just secretory granules. Only a minority of enterocytes might display the hallmarks at the time of EM analysis, and the histological presentation of MVID varies from patient to patient.

Additionally, increasing availability and decreasing costs of modern genetic analysis render it a suitable tool to verify MVID diagnosis. Targeted mutational analysis of the *MYO5B* and *STX3* loci, high-throughput sequencing of genes causing congenital diarrhoeas (‘gene panel’ sequencing) and whole-exome sequencing should be performed.

## Pathophysiology

Over the last years, several studies have added to the understanding of MVID’s pathophysiology [7–10, 15, 20–22]. Besides native biopsy material from MVID patients, the human colorectal adenoma cell line CaCo2 has been used as the model system in most of these studies. As this cell line has the ability to establish enterocyte polarity in culture, it has been the basis for loss of Myo5B studies and its impact on epithelial polarity. This approach allowed to gain further insight into the role of Myo5B in intracellular trafficking as down-regulation and knockout recapitulate the loss of apical microvilli, loss of polarity and the accompanying mislocalisation of apical transporters [8, 9, 20]. This might be a crucial step for understanding MVID, as the mislocalisation of the Na+/H+ exchanger NHE3 [20] might account for the sodium loss diarrhoea reported from MVID cases [23]. The identification of a second gene causal for MVID, the t-SNARE *STX3*, points towards a role of the apical exocytic pathway in epithelial cells. Both Myo5B and Stx3 are involved in apical trafficking [15, 24].

The origin of microvillus inclusions [10, 21] was addressed as well, and hypotheses range from autophagocytosed/endocytosed apical plasma membrane [1, 10, 21, 25] to de novo formed, intracellular apical domains [3, 19]. Conceivably, microvillus inclusions might not add much to the pathophysiology but could rather be a secondary effect [25] of overall disrupted epithelial polarity, since similar structures known as ‘vacuolar apical compartments (VACs)’ also occur in epithelial cancers, or following experimental disruption of the cytoskeleton or of intercellular contacts [26–30].

So far, secretory granules could only rarely be reproduced in in vitro culture [8, 15]. However, a genome-edited CaCo2 cell line was recently published, demonstrating all three ultrastructural hallmarks including an accumulation of subapical vesicles. With this, better understanding of MVID’s pathophysiology was achieved [31].

Only recently, *MYO5B* knockout mouse models have been published. Despite the different genetic approaches applied, constitutive whole organism knockout [32] and inducible bowel-specific knockout [19, 25]demonstrate all these studies MVID-like phenotypes in mice. Schneeberger and colleagues demonstrated that organoid cultures of murine intestinal stem cells might serve a useful tool to test future therapies for MVID [19]. For this, crypt stem cells obtained by endoscopic biopsies are cultured to form ‘mini-guts’ ex vivo.

## Outlook

The increase in understanding the pathophysiology of MVID will eventually allow developing targeted therapies to treat specific symptoms, e.g. diarrhoea, and could overcome the need to transplant the small bowel and perform long-term parenteral nutrition. This could lead to a drastic increase in the quality of life of MVID patients. New therapeutic approaches could be tested on organoid cultures derived from mice or patients. This, of course, remains speculative at the moment, but conceivable targets of therapy could be the disrupted epithelial polarity and the accompanying mislocalisation of apical enzymes and transporters.

In recent years, novel genome editing technologies, such as CRISPR/Cas9, once again moved gene therapy into the spotlights. Enterocyte stem cells reside at the bottom of every crypt in the bowel [33]. Thus, all these stem cell niches have to be targeted in order to achieve therapeutic efficiency. Besides all still unsolved problems of genome editing, e.g. off targets [34], crypt stem cells niches make gene therapy rather unsuitable for MVID.

## Abbreviations

EM: electron microscopy
MLPA: multiplex ligation-dependent probe amplification
MVID: microvillus inclusion disease
PAS: periodic acid-Schiff
SNARE: *N*-ethylmaleimide-sensitive factor attachment protein receptor
TPN: total parenteral nutrition
Tx: transplantation
